# The oscillatory flow of the cerebrospinal fluid in the Sylvian aqueduct and the prepontine cistern measured with phase contrast MRI in children with hydrocephalus—a preliminary report

**DOI:** 10.1007/s00381-017-3699-0

**Published:** 2018-01-10

**Authors:** Emilia Nowosławska, Dominika Gwizdała, Dobromiła Barańska, Piotr Grzelak, Michał Podgórski, Krzysztof Zakrzewski, Bartosz Polis, Mariusz Stasiołek, Lech Polis

**Affiliations:** 1 Department of Neurosurgery, Polish Mother’s Memorial Hospital Research Institute, Łódź, Poland; 2 Department of Diagnostic Imaging, Polish Mother’s Memorial Hospital Research Institute, Łódź, Poland; 3 Department of Neurology, Polish Mother’s Memorial Hospital Research Institute, Łódź, Poland

**Keywords:** Brain compliance, Hydrocephalus, Peak velocity coefficient

## Abstract

**Introduction:**

Recognizing patients with ventriculomegaly who are at risk of developing acute hydrocephalus presents a challenge for the clinician. The association between disturbed cerebrospinal fluid flow (CSF) and impaired brain compliance may play a role in the pathogenesis of hydrocephalus. Phase contrast MRI is a noninvasive technique which can be used to assess CSF parameters. The aim of the work is to evaluate the effectiveness of phase contrast MRI in recognizing patients at risk of acute hydrocephalus, based on measuring the pulsatile CSF flow parameters in the Sylvian aqueduct and prepontine cistern in children with ventriculomegaly.

**Aim:**

The aim of the work is to characterize the parameters of cerebrospinal fluid (CSF) flow in the Sylvian aqueduct and prepontine cistern in children with ventriculomegaly with regard to patient age and symptoms. We hypothesize that the relationship between CSF flow parameters in these two regions will vary according to analyzed factors and it will allow to recognize children at risk of hydrocephalus.

**Materials and methods:**

A group of 26 children with ventriculomegaly (five girls and 21 boys) underwent phase contrast MRI examinations (Philips 3T Achieva with Q-flow integral application). Amplitudes of average and peak velocities of the CSF flow through the Sylvian aqueduct and prepontine cistern were used to calculate ratios of oscillation and peak velocities, respectively. The relationship between the oscillation coefficient, the peak velocity coefficient, and stroke volume was then assessed in accordance with age and clinical symptoms.

**Results:**

The peak velocity coefficient was significantly higher in patients with hyper-oscillating flow through the Sylvian aqueduct (3.04 ± 3.37 vs. 0.54 ± 0.28; *p* = 0.0094). Moreover, these patients tended to develop symptoms more often (*p* = 0.0612). No significant age-related changes were observed in CSF flow parameters.

**Conclusion:**

Phase contrast MRI is a useful tool for noninvasive assessment of CSF flow parameters. The application of coefficients instead of direct values seems to better represent hemodynamic conditions in the ventricular system. However, further studies are required to evaluate their clinical significance and normal limits.

## Introduction

The key point to understand the nature of hydrocephalus lies in understanding the nature of CSF circulation. This issue has been broadly studied since 1940 [27]. Nevertheless, a lack of proper imaging techniques resulted in the utilization of animal models [4, 19, 30]. It is not certain whether derived conclusions correspond to hydrodynamics of human CSF; however, vital kernels of knowledge can be obtained by finding new ways to apply imaging techniques.

The phase contrast MRI technique, invented in the 1980s, allows for the noninvasive evaluation of the CSF flow. It does not require a contrast administration because this technique assumes that atomic spins moving in the same direction as a magnetic field gradient produce a phase shift that is proportional to the velocity of these spins. Previous applications of the phase contrast MRI have demonstrated that the oscillatory character of CSF flow is consistent with the cardiac cycle and respiratory rate [2, 4, 6, 11, 23]. This observation called into question some aspects of the traditional CSF bulk flow theory created by Dandy and Blacfan in 1914, which does not fully explain the genesis of communicating hydrocephalus [6]. Furthermore, this theory contradicts Pascal’s law, which states that the transmantle pressure gradient cannot be the main force leading to ventricular enlargement [24, 26]. In fact, a transmantle pressure gradient, albeit relatively small, is detected even in healthy people. In people with hydrocephalus, this pressure is slightly higher than in healthy people (0.62 vs. 0.56 mmHg, respectively). This could be explained by the fact that CSF is not a static body but a dynamic liquid, which can be described by Laplace’s law [16].

The Linninger mathematical model of CSF hydrodynamics is more congruent with data obtained from the phase contrast MRI than traditional concepts. According to hydrodynamic theory, the main cause of hydrocephalus is the loss of brain compliance and elasticity, resulting in development of an increased transmantle pulsatile gradient between the intraventricular lumen and subarachnoid spaces. Studies have supported this theory only in small groups of adults [1, 4, 6–8, 11–13, 15, 16, 19, 25, 27, 28, 30]. No analogous data exists for children. Acquiring such data for pediatric patients would be particularly valuable partly because hydrocephalus occurs more frequently in children than in adults and partly because the data could be used as a benchmark for pediatric patients concerning the suspected changes in the CSF hydrodynamics that occur with maturation [5, 8, 21, 28].

## Clinical material and methodology

The study group comprised 26 children (five girls and 21 boys) with ventriculomegaly or at risk of hydrocephalus because of completed intraventricular hemorrhage. Patient age ranged from 7.2 months to 12 years, on average 3.5 years (± 3.05 years). The most common causes of ventriculomegaly were idiopathic, the presence of a cyst interfering with physiological CSF flow, or intraventricular bleeding. The exclusion criteria comprised any brain malformations not associated with ventriculomegaly, aqueduct occlusion, or cerebral shunt implantation. The study protocol was approved by the ethics committee (protocol number-95/2015).

**Fig. 1:**
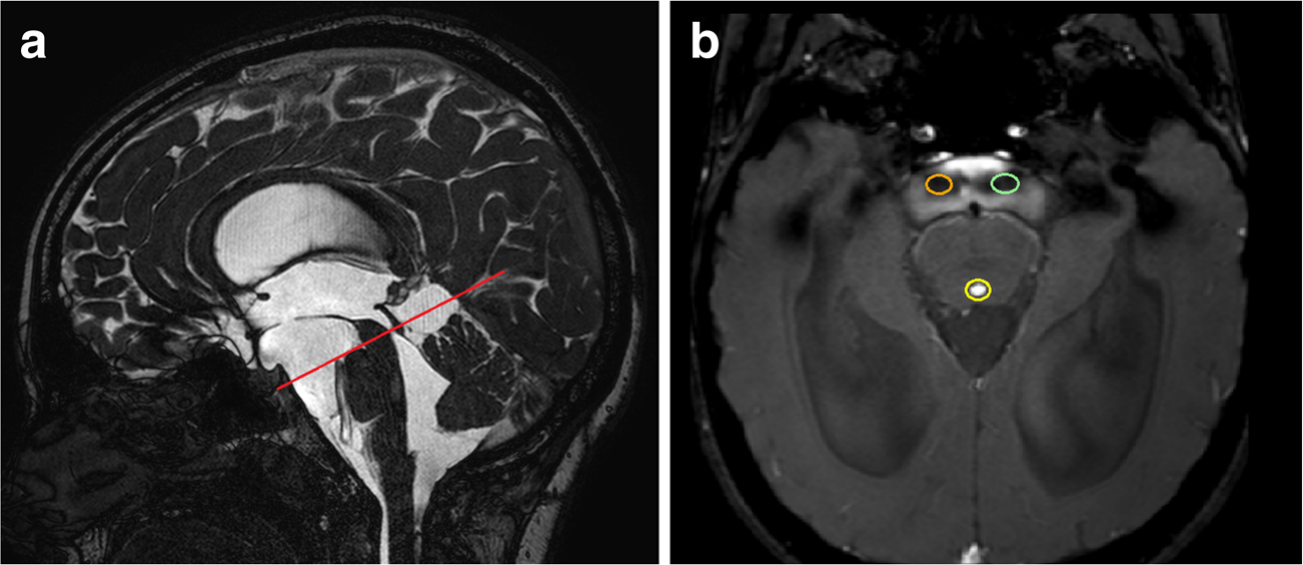
**a** Sagittal scan of CSF BFFE sequence used to plan the CSF QF sequence plane (red line). **b** Placement of ROIs in the aqueduct (yellow ellipse no. 1) and in two alternative places in the prepontine cistern (green ellipse no. 2 and orange ellipse no. 3)

The patients underwent the phase contrast MRI examinations (Philips 3T Achieva) according to the following protocol. ECG electrodes were placed in standard positions and ECG trace was obtained. First, the structural brain images were acquired. CSF BFFE sequence sagittal scans were used to determine the plane for the CSF QF sequence (TR/TE, 22/14; matrix, 220 × 154; voxel 0.68/0.97/4, FOV HF 200 x AP 230, acquisition time ≈ 3 min). The CSF QF sequence plane was positioned perpendicular to the Sylvian aqueduct and prepontine cistern at the level of the inferior colliculi of the quadrigeminal plate (Fig. 1a). Cine loops of CSF flow during consecutive cardiac cycles were acquired. The velocity encoding value was 0–20 cm/s. Further analysis was performed with Q-flow software (Intellispace Portal, Philips Healthcare). Two regions of interests (ROIs) were drawn manually, encompassing all pixels that reflected the CSF flow signals of the aqueduct (ROI 1) and part of the prepontine cistern with the most vigorous flow, as far as possible from the neighboring basilar artery (ROI 2) (Fig. 1b).

MRI examination was used to evaluate the amplitudes of the average velocities and peak velocities of the CSF flow over cardiac cycles in the Sylvian aqueduct and prepontine cistern. To analyze oscillatory CSF flow, the ratio of amplitudes in the aqueduct and the prepontine cistern, the so-called *oscillation coefficient*, was calculated. The CSF flow through the aqueduct was recognized as hyper-oscillatory if the oscillation coefficient was ≥ 1 [17, 18]. The ratio of peak velocities was calculated in a similar manner and termed *peak velocity coefficient*. Another CSF flow parameter measured in the Sylvian aqueduct was the stroke volume: the volume of CSF flowing through the aqueduct forward and backward during a cardiac cycle. Brain compliance was regarded as diminished when the stroke volume, representing the compliance and elasticity of the brain, was ≥ 42 μl [6].

CSF flow parameters were analyzed in the context of patient age and symptoms. As a pattern of the CSF flow is considered to be immature in children ≤ 2 years, but the same as in adults in older children [5], the patients were divided into two groups: infants and toddlers aged up to 2 years (10 children) and those aged between 3 and 18 years (16 children). The CSF flow parameters were also compared between symptomatic and asymptomatic patients due to the fact that some of them developed the following clinical symptoms: epileptic seizures (*n* = 13), headaches (*n* = 4), paresis (*n* = 17), mental retardation (*n* = 13), the increased head circumference growth rate (*n* = 15), signs of chronic increased intracranial pressure (*n* = 2). Eight children did not show any clinical symptoms (Table 1).

**Table 1:** Symptoms: 1—increased head circumference growth rate; 2—paresis; 3—signs of chronic increased intracranial pressure; 4—mental retardation; 5—headaches; 6—epileptic seizures

Pt.	Age [years]	Diagnosis	FOHR	Symptoms	Symptoms duration [years]
1	6	Dandy-Walker syndrome variant	0,6	1, 2, 4, 6	6
2	0,6	Congenital ventriculomegaly	0,47	–	–
3	0,75	Congenital ventriculomegaly	0,39	1, 2, 4, 5	0,75
4	4	Intraventricular hemorrhage	0,43	–	–
5	0,6	Intraventricular hemorrhage	0,37	–	–
6	2,84	Congenital ventriculomegaly	0,53	1, 2, 4, 6	2,84
7	2	Intraventricular hemorrhage	0,35	–	–
8	6	Intraventricular hemorrhage	0,53	1, 2, 3, 4, 6	6
9	2	Congenital ventriculomegaly	0,43	1, 2, 6	2
10	3	Congenital ventriculomegaly	0,46	1, 2, 4, 6	3
11	4	External benign hydrocephalus	0,35	5	4
12	2	Chiari	0,57	1, 2, 4, 6	2
13	2	Intraventricular hemorrhage	0,49	–	2
14	1,75	Intraventricular hemorrhage	0,45	1, 2, 4, 6	1,75
15	3,5	Dandy-Walker syndrome variant	0,43	2, 4	3,5
16	2,5	Suprasellar arachnoid cyst	0,63	1, 2, 5, 4, 6	2,5
17	5,75	Congenital ventriculomegaly	0,45	3, 2, 5, 6	5,75
18	1,58	Congenital ventriculomegaly	0,6	1, 2, 4, 6	1,58
19	2,25	Congenital ventriculomegaly	0,45	1, 2	2,25
20	11	Congenital ventriculomegaly	0,51	1, 2, 4, 6	11
21	1	Intraventricular hemorrhage	0,56	1, 2, 4, 6	1
22	12	Congenital ventriculomegaly	0,45	–	12
23	7	Congenital ventriculomegaly	0,55	1, 2, 4, 6	7
24	14	Chiari	0,5	–	14
25	4	Congenital ventriculomegaly	0,17	1, 2	4
26	7	Congenital ventriculomegaly	0,53	–	7

Statistical analysis was performed using Statistica 12 software (StatSoft Poland, Cracow, Poland). Although a *p* level of < 0.05 was generally considered significant, the Bonferroni correction was added and the level of significance lowered to < 0.017 for multiple testing. For between-group comparisons of nominal variables, the chi^2^ test with dedicated corrections was applied. The normality of continuous data distribution was checked with the Shapiro-Wilk test. Due to skewed distribution of CSF flow parameters within each analyzed subgroup, the Mann-Whitney test was applied for comparisons.

## Results

**Fig. 2:**
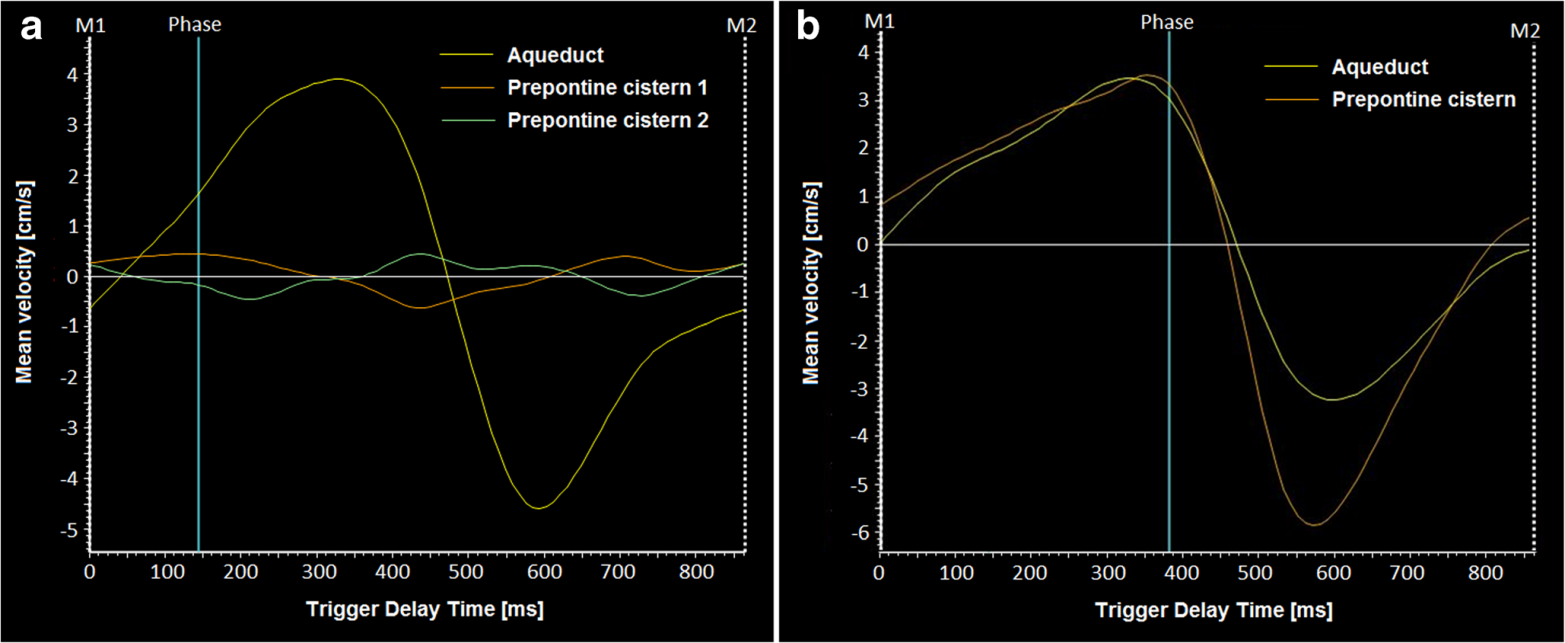
**a** An example of mean velocity plot for a patient with hydrocephalus and clinical symptoms (patient presented in Fig. 1b) shows high amplitude of flow velocity in the aqueduct (line no. 1) and low amplitude in the prepontine cistern (line nos. 2 and 3). Two ROIs were placed in the prepontine cistern to show that side of ROI placement (left or right to the basilar artery) results in almost the same line on the plot (line no. 3 is inverted to better separate from line no. 2). **b** An example of patient without clinical symptoms, who has high flow velocity amplitude in both aqueduct (line no. 1) and prepontine cistern (line no. 2)

No significant differences were observed in any of the analyzed CSF flow parameters with regard to the presence of clinical symptoms, age, or decreased brain compliance (stroke volume ≥ 42 μl) (Table 2). However, hyper-oscillating flow through the Sylvian aqueduct (oscillation coefficient ≥ 1) was observed more frequently in patients affected by clinical symptoms (12 individuals) than in patients with no symptoms (two individuals). This relationship was on the verge of significance (*p* = 0.0612) (Fig. 2). Moreover, the peak velocity coefficient was significantly higher in the group with hyper-oscillating flow (14 individuals with hyper-oscillating flow, mean value 3.04 ± 3.37 vs. 12 individuals with normal flow, mean value 0.54 ± 0.28; *p* = 0.0094).

**Table 2:** Comparison of CSF flow parameters according to symptoms, age, and brain compliance

	Oscillation coefficient mean (SD)	Peak velocity coefficient mean (SD)	Stroke volume [μl] mean (SD)
Symptoms present (*n* = 18)	2.29 (3.23)	3.92 (2.26)	30.05(34.50)
No symptoms (*n* = 8)	0.98 (0.59)	2.83 (1.18)	6.4(6.16)
*p* value	0.2216	0.3449	0.09
Age ≤ 2 years (*n* = 10)	1.58 (1.27)	4.22 (2.18)	13.98 (9.61)
Age > 2 years (*n* = 16)	2.07 (3.40)	3.19 (1.89)	29.39 (37.79)
*p* value	0.8744	0.2918	0.9370
Stroke volume < 42 μl (*n* = 21)	1.29 (1.01)	3.35 (1.94)	
Stroke volume ≥ 42 μl (*n* = 5)	4.37 (5.72)	4.56 (2.34)	
*p* value	0.1929	0.2830

As far as age was concerned, no significant difference was found between the mean age of children with hyper-oscillating flow (14 patients, 4.5 ± 3.9) and of children with oscillation coefficient < 1 (12 patients, 3.9 ± 3.2) (*p* = 0.938). The theoretical age of the change in CSF hemodynamic pattern (2 years of age) did not affect the frequency of the hyper-oscillating flow occurrence: for children aged over 2 years, eight had an oscillation coefficient < 1 while six had an oscillation coefficient ≥ 1; for those aged 2 or less, six had an oscillation coefficient ≥ 1 while four had an oscillation coefficient < 1 (*p* = 0.4640).

No significant relationship was found between decreased brain compliance (stroke volume ≥ 42 μl) and age or with CSF hemodynamics and age (Tables 2 and 3).

**Table 3:** Comparison of age and oscillation index according to brain compliance

	Stroke volume < 42 μl	Stroke volume ≥ 42 μl	*p* value
Age of children in years [Mean (SD)]	4.1 (3.9)	4.5 (1.4)	0.2549
Age > 2 years	11	5	0.1213
Age ≤ 2 years	10	0	
Oscillation coefficient ≥ 1	10	4	0.3304
Oscillation coefficient < 1	11	1	

### Illustrative case

Neuroendoscopic ventriculo-cystocysternostomy was performed in one patient due to the presence of a suprasellar arachnoid cyst that impaired outflow of the CSF from the third ventricle. Due to increasing head circumference, MRI examination was performed. Both the oscillation coefficient (14.38) and the peak velocity coefficient (7.7) were relatively high in comparison with the rest of the group. The measured stroke volume was 54 μl indicating decreased brain compliance. Based on clinical presentation and MRI results, the patient was qualified for shunt implantation. The procedure was performed with no complication and resulted in clinical improvement.

## Discussion

A key novel aspect of this study is that in contrast with the majority of similar studies, which deal with adults, it concerns children with ventriculomegaly. Secondly, instead of assessing the direct parameters of CSF flow in the Sylvian aqueduct, we analyzed coefficients, including the flow through the subarachnoid space, thus giving a better insight into the equilibrium between the ventricular system and the subarachnoid space.

The study has three central findings. In children with ventriculomegaly, hyper-oscillation of the CSF flow through the Sylvian aqueduct is associated with significantly greater peak flow velocity. Secondly, there is a trend in symptomatic children to develop a hyper-oscillating type of flow. Finally, age-related changes of the pattern of CSF hemodynamics may be disturbed in patients with ventriculomegaly.

Understanding the dynamics of the movement of CSF appears a key for proper characterization of the pathomechanism of hydrocephalus. CSF flow is influenced by the blood pressure wave over the cardiac cycle and by the respiratory rate to a lesser extent [30]. According to Linninger, hydrocephalus is characterized by the decreased compliance of the brain arterial system and subarachnoid space, as well as by decreased arterial blood inflow [1, 4, 7, 8, 11–13, 15–18, 25–28, 30]. This model concerns the brain as a poroelastic substance, where increased stiffness, i.e., decreased compliance, results in excessive fluid storage due to decreased fluid outflow [9, 17, 18]. The water content in the subependymal areas of the brain is known to be elevated in patients with normal pressure hydrocephalus (NPH), as demonstrated by diffusion MRI [22]. This is in accordance with the *microvessels theory*, hypothesizing that CSF flow not only manifests as a *bulk flow* from the choroid plexus to the arachnoid villi, but also as a free admixing between the subarachnoid space and extracellular space (exchange across capillary beds in situ, without a bulk flow). This exchange is controlled by the glymphatic system [5, 8, 26] and by the activity of Aquaporion 4 (AQP4) in the ependyma and endothelium [26, 28].

The creation of a precise CSF hydrodynamics model remains a challenge. A transmantle pressure gradient exists both in healthy volunteers and patients affected by hydrocephalus, and although the pressure is slightly higher in the hydrocephalus patients, it is still too low to be the main force responsible for driving the CSF movement [18, 19, 29, 30].

Phase contrast MRI was first performed in 1980, when Holland observed the disappearance of an MRI signal associated with the oscillating movements of CSF molecules [2, 4, 6]. The caudal part of the Sylvian aqueduct is suitable for CSF measurement because it has a tubular shape and CSF flow in this localization has a laminar character [20].

The most popular and reliable parameter for assessing CSF is a stroke volume, which represents the volume of CSF flowing back and forth during the cardiac cycle [14]. Despite the best efforts of researchers, a large degree of variance in stroke volume has been found between studies. The limit values of stroke volume for diagnosing hydrocephalus also vary greatly. Bradley et al. claimed that the stroke volume ≥ 42 μl is characteristic of active hydrocephalus which responds to shunt treatment. Other authors have stated that this value could be as high as 71 or 138 μl [2, 6, 10, 11, 30]. However, 42 μl was assumed as the upper limit for proper elasticity and compliance for the purposes of the present study as it was the lowest limit given in the literature, and direct CSF flow parameter values are lower in children than in adults.

A great degree of variation has been found in CSF flow parameters depending on the study: the upper limit of the flow rate ranges from 18 to 24 ml/min [2, 3, 11], and the upper limit of peak flow velocity from 9 to 10 cm/s [24, 25]. Hence, the raw CSF flow values seem not to be a reliable and strong diagnostic criteria for hydrocephalus. However, an alternative approach based on proportions (coefficients) of these parameters may be a solution. While healthy adults have been found to demonstrate higher peak flow velocity in the prepontine cistern (on average 13.93 ± 9.97 mm/s, *N* = 6) than the Sylvian aqueduct (on average 3.82 ± 2.12 mm/s.), the opposite is true for patients with NPH (10.82 ± 1.16 mm/s in the prepontine region and 13.14 ± 2.64 mm/s in the Sylvian aqueduct). Hydrodynamic theory assumes that decreased compliance between the arterial system and subarachnoid space results in greater pulsation of the brain parenchyma and the formation of a water hammer effect; this causes a transmantle oscillatory gradient resulting in the enlargement of the cerebral ventricles. Therefore, the coefficient of CSF flow through the subarachnoid space and the Sylvian aqueduct may be a better indicator of decreased brain elasticity than the absolute peak velocity flow value [17, 18]. Published data indicates that the peak velocity coefficient between the Sylvian aqueduct and prepontine cistern should be about 0.3, rising to about 1.2 in adult patients with NPH. In our patients, these values were higher: 3.92 ± 2.26 for symptomatic patients and 2.83 ± 1.18 for asymptomatic ones. In addition, the children with clinical symptoms examined in the present study were more likely to demonstrate hyper-oscillating flow through the Sylvian aqueduct, although this trend was of borderline significance (*p* = 0.0612). Both these findings may be signs of increased CSF absorption resistance. This hypothesis is best represented in the illustrative case of our patient with ventriculo-cystocysternostomy, who presented a high oscillation coefficient (14.38) and peak velocity coefficient (7.7), together with a stroke volume of 54 μl, indicating decreased brain compliance.

Other explanation for observed differences in CSF flow parameters between children and adults may result from distinct characteristics of the CSF hydrodynamics. Arachnoid villi are fully developed in the fetus before birth, but they only begin functioning at late infancy. Before then, in neonates, infants, and young children, the main role in CSF absorption plays the interstitial and perivascular spaces as well as perineural lymphatic channels (so-called minor CSF pathways). It has also been observed that in heathy children younger than 2 years, CSF mainly returns to the cerebral ventricular system, which indicates that CSF absorption takes place through the minor CSF pathways. The opposite situation takes place in older children and adults [5]. Although our findings do not reveal any significant differences in flow parameters between particular age groups, this may be due to the fact that the described CSF flow model concerns healthy children. Therefore, our observations do not contradict this theory of changes in CSF hydrodynamics in children, but that CSF circulation through minor pathways in children is as effective as circulation through the macro CSF pathways in adults [21, 28].

Our most interesting finding was lack of any significant relationship between the marker of brain elasticity and hyper-oscillating flow in the aqueduct. This could be the result of an inappropriate limits assumed for brain elasticity. Perhaps, the stroke volume of ≥ 42 μl assessed by Bradley as significant for the adult patients who had lost brain compliance does not apply to children. Alternatively, more samples could be needed for a more accurate analysis: the group with the stroke volume ≥ 42 μl only comprised five children. However, a significant relationship was found between the oscillating coefficient and the flow velocity coefficient. The two measurements should not be treated as associated data because the oscillating coefficient describes the character of CSF flow rather than its absolute speed.

There are two major limitations of this study. The first is that the study sample was too small to provide sufficient power. However, our aim was to present a new perspective for assessing CSF flow parameters as coefficients for the assessment of dynamic equilibrium rather than as direct values in a particular location. The second limitation was the lack of a control group. However, due to the fact that most children in the study were under 6 years old, the lower limit for performing MRI examination without sedation/anesthesia, it was not justifiable to expose healthy children to potentially harmful side effects. In future, we plan to gather a larger sample of patients and analyze the differences in CSF flow parameters between patients who require intervention, as in our illustrative case, and those who remain in a stable condition. It is also worth to mention that application of the phase contrast MRI may be associated with technical limitation, concerning placement of the ROI in the prepontine cistern. Particularly, when this space is narrow or scarred (e.g., post-hemorrhagic scarring). In these cases, adjustment of the ROI size and its placement in some distance from the basilar artery are vital and, according to our experience, possible in almost all cases.

### Summing up

The peak velocity coefficient was significantly higher in children with hyper-oscillating CSF flow in the Sylvian aqueduct. Moreover, this type of flow occurred more often in symptomatic children. Finally, probably, due to ventriculomegaly, the physiological maturation of CSF flow pattern was disturbed. However, diagnostic limits of CSF flow parameter values in patients affected by neurological symptoms require further research based on larger groups with a control group composed of healthy children with normal-size ventricular system.
